# Inbreeding does not alter the response to an experimental heat wave in a freshwater snail

**DOI:** 10.1371/journal.pone.0220669

**Published:** 2019-08-08

**Authors:** Katja Leicht, Jukka Jokela, Otto Seppälä

**Affiliations:** 1 Department of Aquatic Ecology, Swiss Federal Institute of Aquatic Science and Technology, Dübendorf, Switzerland; 2 Department of Biological and Environmental Science, University of Jyväskylä, Jyväskylä, Finland; 3 Institute of Integrative Biology (IBZ), ETH Zürich, Zürich, Switzerland; 4 Research Department for Limnology, University of Innsbruck, Mondsee, Austria; Natural History Museum of London, UNITED KINGDOM

## Abstract

Global climate change affects natural populations of many species by increasing the average temperature and the frequency of extreme weather events (e.g. summer heat waves). The ability of organisms to cope with these environmental changes can, however, depend on their genetic properties. For instance, genetic load owing to inbreeding could alter organisms’ responses to climate change-mediated environmental changes but such effects are often overlooked. We investigated the effects of an experimental heat wave (25°C versus 15°C) on life history (reproduction, size) and constitutive immune defence traits (phenoloxidase-like and antibacterial activity of haemolymph) in relation to inbreeding by manipulating the mating type (outcrossing, self-fertilization) in two populations of a hermaphroditic freshwater snail, *Lymnaea stagnalis*. High temperature increased reproduction and size of snails but impaired their immune function. In one of the two study populations, inbreeding reduced reproductive output of snails indicating inbreeding depression. Furthermore, this effect did not depend on the temperature snails were exposed to. Our results suggest that *L*. *stagnalis* snails can be negatively affected by inbreeding but it may not alter their responses to heat waves.

## Introduction

Owing to global climate change, the average temperatures at the Earth’s surface, as well as the frequency and severity of extreme weather events, are increasing [1, 2]. Of these, extreme weather conditions such as summer heat waves are suggested to be more significant to natural populations than the gradual increase in average temperatures [3, 4]. This is because high temperatures can have strong effects on organismal physiology and even alter species interactions [5–8]. Such effects are largely due to changes in fitness-related traits such as reproduction [9, 10] and defence against natural enemies [11, 12]. The responses of organisms to environmental alterations can, however, vary across individuals and populations depending on their genetic properties such as allele composition and the level of inbreeding [13–15].

Self-fertilization and reproduction with related individuals (i.e. biparental inbreeding) that are often seen in small populations both increase genome-wide homozygosity of organisms [16]. Increased homozygosity can lead to the expression of recessive deleterious alleles (i.e. genetic load) that can reduce fitness by altering fitness-related traits [17, 18]. This is known as inbreeding depression. In previous research, inbreeding has been shown to affect organisms’ responses to pollution, desiccation, food deprivation, and temperature extremes indicating inbreeding-by-environment interactions [13, 19]. This could be because of the context-dependent expression of the genetic load [20, 21]. However, the potential interaction between anthropogenic climate change and inbreeding that often results from human-induced decrease in population size is rarely examined (but see [22, 23, 24]).

We investigated the combined effects of an experimental heat wave and inbreeding on life history traits (reproduction and size) and constitutive immune defence traits [phenoloxidase (PO)-like activity and antibacterial activity of haemolymph] in a simultaneously hermaphroditic freshwater snail, *Lymnaea stagnalis*. All these traits contribute to snail lifetime fitness [25]. Additionally, immune defence can be highly important under climate change as the risk of infections is predicted to increase under high-temperature conditions [26, 27]. At a benign temperature, inbreeding is known to reduce the growth [28] but not the immune function and parasite resistance [28, 29] of *L*. *stagnalis*. Furthermore, exposure to high temperature (≥ 25°C) initially increases snail growth and reproduction [30–32], but this leads to reduced immune function [30, 31, 33]. Here, we tested whether the above effects interact by experimentally manipulating the mating type (outcrossing, self-fertilization) of snails. We predicted inbred snails to be more prone to the negative effects of high temperature and not to be able to increase their growth and reproductive output during the heat wave.

## Materials and methods

### Ethics statement

This study was carried out in accordance with the laws governing animal experimentation in Switzerland, where work with snails does not require permissions. The study did not involve endangered or protected species. No specific permits were required for the field operations as the used water bodies are not private property or a nature reserve.

### Experimental animals

*Lymnaea stagnalis* is a hermaphroditic snail that inhabits lakes and ponds in the Holarctic region. It is a host for various parasites including several trematode species [34] that castrate the snails and increase their mortality [35]. The snails we used in this experiment came from two laboratory stock populations (F_4_ generation) that originated from two different ponds in Zürich, Switzerland (Zürichberg: 47°23'N, 8°33'E; Adlisberg: 47°22'N, 8°34'E). The ponds are 2.7 km apart from each other and unconnected. The genetic analyses of the individuals used as a parental generation in the study (see the section about genetic analyses below) revealed that the stock populations showed genetic polymorphism (S1 Table) typical for natural populations in northern Switzerland [36] but they were genetically differentiated from each other [48% of the alleles were observed only in one of the examined populations (S1 Table); F_st_ = 0.094]. In the study area, the water temperature in ponds is typically low (< 16°C) during summers, although it depends on pond hydrology [T. Salo, unpublished data]. Heat waves, however, can rapidly increase the temperature to 20–30°C, and it can remain high for over two weeks [T. Salo, unpublished data].

We started the stock populations by collecting 45 adult snails from each pond. As *L*. *stagnalis* prefers outcrossing [37, 38] and can store allosperm from matings for over two months [39] offspring of these snails should representatively capture the genetic variation in the source populations. We maintained each stock population with a population size of approximately 400 snails to avoid loss of genetic polymorphism due to genetic drift [40] and kept the snails at a water temperature of 15 ± 2°C, which was also the control temperature used in the experiment. Under these conditions, both stock populations preferred outcrossing over self-fertilization (S1 Table). This allowed manipulation of inbreeding in experimental snails using mating treatments as described below.

We haphazardly collected 40 snails from each stock population to use them as parents of experimental individuals. We collected these snails as juveniles (shell length < 15 mm). We isolated them in perforated plastic cups (200 ml) sunk into a water bath (15 ± 1°C) that was connected to a biological filter. We fed the snails with fresh lettuce ad libitum and maintained them until they started to lay self-fertilized eggs. Then, we randomly assigned the snails into different mating treatments. We used one-third of the snails that were still alive to produce self-fertilized families by maintaining them individually in their cups (i.e. forced self-fertilization). We formed pairs from the rest of the snails to give them an opportunity to mate and produce outcrossed offspring. We kept the snails from each pair together in a cup for two days and isolated them after that. This allowed separating clutches laid by different individuals within each pair. After the matings were completed, we collected the first clutch containing more than 30 eggs from each self-fertilizing snail (seven individuals in the Zürichberg population and six individuals in the Adlisberg population laid eggs) as well as from one snail within each outcrossing pair (seven individuals per population laid eggs) to produce the experimental snails. All the egg clutches were collected within a week.

We maintained the collected egg clutches individually in plastic containers with 6 l of aged tap water at 15 ± 1°C. We maintained the hatched snails in family groups in the same containers (20 to 30 individuals per container) and fed them with Spirulina powder until they reached a shell length of approximately 5 mm. Then, we fed the snails with fresh lettuce ad libitum. We changed the water in the containers once a week. We started the experiment (see the next section) after eight weeks, which was when the snails had reached maturity (shell length > 20 mm).

### Experimental design

We randomly assigned experimental snails from each family into two different temperature treatments (25 ± 1°C and 15 ± 1°C; 15 snails per family per treatment; a total of 810 snails). We chose 25°C as the high temperature as it lies above the thermal optimum of snails [41] and occurs intermittently in the habitats of snails during hot summers [T. Salo, unpublished data]. We used 15°C as the control temperature as it is close to the thermal optimum of snails [41] and common in ponds [T. Salo, unpublished data]. We isolated the snails into cups filled with aged tap water at 15°C and transferred them to the experimental temperatures. This allowed a slow (1°C h^-1^) change to the target temperature for snails exposed to the experimental heat wave. We then transferred each snail into a perforated plastic cup (200 ml) and sunk the cups into similar water baths as above. We exposed the snails to their respective temperature treatments for seven days. We chose one-week exposure as it represents a typical heat wave in Western Europe (average: 8.4 days [1]) and is known to affect snail reproduction and immune defence (increased fecundity at the expense of immune function; see references above). After the 7-day exposure period, we measured the shell length (to the nearest 0.1 mm), total reproductive output during the experiment, and immune function of each snail (see the section about measurements below). We conducted the experiment in three blocks that started on consecutive days. Each block had three to five snails per family per temperature treatment.

### Measurements

To estimate the reproductive output of snails, we collected all the egg clutches laid during the experiment. It is important to note that the experimental snails had had an opportunity to mate only with close relatives (i.e. siblings). However, this should not reduce reproductive output as even self-fertilization has not been reported to influence snail fecundity [37]. We placed the collected egg clutches on a millimeter paper and photographed them from 10 cm above with a Fujifilm FinePix F30 digital camera (scene mode: close up, focal length: 35 mm, aperture: F/2.8, shutter speed: 1/85, sensitivity: ISO-200, image size: 2848 × 2136 pixels, focus mode: autofocus). From each picture, we measured the two-dimensional area of the clutch and the area containing ten eggs using ImageJ (ImageJ 1.42q, Wayne Rasband, National Institute of Health, USA). We then calculated an estimate of the total number of eggs in the clutch (the area of the clutch divided by the area containing ten eggs multiplied by ten). We summed up the number of eggs laid by each snail for the estimate of total reproductive output.

To quantify snail immune activity we measured two immune parameters, phenoloxidase (PO)-like activity and antibacterial activity of haemolymph. Phenoloxidase enzymes belong to oxidative defences used against eukaryotic parasites [42] whereas antibacterial proteins reflect the humoral response against microorganisms [43]. These parameters are central in the immune system of invertebrates, including molluscs [44, 45]. In *L*. *stagnalis*, they respond to various immune elicitors [46] and are subject to natural selection [25]. We measured snail immune activity rather than susceptibility to any specific parasite as the latter type of studies are necessarily specific to the chosen parasite species and can be confounded by the direct effects of temperature on parasite transmission stages [47, 48].

We measured the immune parameters as described in [30]. Briefly, we collected snail haemolymph by gently tapping the undersides of their feet with a pipet tip until they retreated into their shells, simultaneously releasing haemolymph through the hemal pore [49]. This is a normal antipredatory response in this species [50]. We measured PO-like activity and antibacterial activity of haemolymph spectrophotometrically using a microtiter plate reader (SpectraMax 190, Molecular Devices, Sunnyvale, CA, USA). For the measurements of PO-like activity, we mixed haemolymph with L-Dopa and measured the increase in the optical density (OD) of the solution. This is due to the oxidization of L-Dopa by PO. For the measurements of antibacterial activity, we mixed haemolymph with lyophilized *Escherichia coli* cells and measured the decrease in OD, which is due to the lysis of the bacteria cells.

### Genetic analyses to verify outcrossing/self-fertilization

We tested whether the families produced by the snails maintained together with a mating partner were outcrossed or self-fertilized using 12 microsatellite loci {GenBank Accession No. AY225956-AY225959, AY225961-AY225963, EF208747-EF208749, EF208751, and EF208752 [Knott et al. 2003, K. C. Kopp & Wolff, K., unpublished data (direct submission to GenBank)]}. We conducted the analysis as described in [15] except for the following modifications. In multiplex reaction 1, we added 0.2 (F + R) μM of primer 2k33, 0.4 μM of primer D5, and 0.8 μM of primer B117 and 2k46. In multiplex reaction 2, we added 0.2 (F + R) μM of primer 2k42 and B4, 0.4 μM of primer A112, and 0.6 μM of primer A102. In multiplex reaction 3, we added 0.2 (F + R) μM of primer A16, 0.4 μM of primer 2k27 and C4, and 0.6 μM of primer 2k11.

To verify outcrossing/self-fertilization we first identified loci in which the parents of each family carried different alleles. Then, we amplified these loci in 10 to 15 experimental snails per family to see whether they carried one allele from each parent indicating outcrossing. We did not genotype all experimental snails because *L*. *stagnalis* typically produces all offspring in the same egg clutch through either outcrossing or self-fertilization [28]. In these mating pairs, four of the families were produced through outcrossing and three through self-fertilization in each population. Thus, the final mating type categories for the used families were (1) produced through outcrossing (4 families per population), (2) produced through forced self-fertilization (Zürichberg: 7 families; Adlisberg: 6 families), and (3) produced through self-fertilization despite access to a mate (3 families per population). Because self-fertilizing in the presence of a mate can be a decision based on, for instance, the condition of the mating partner we treated the two groups of snails produced through self-fertilization separately.

### Statistical analyses

Snail survival could not be used as a response variable in the analyses because there was no mortality during the experiment. Furthermore, not all examined traits could be measured from 15 snails (1.9% of all individuals). This was because of an insufficient amount of haemolymph or human errors during the measurements and we excluded these individuals from the data. We analysed the variation in the probability of snails to lay eggs during the experiment using a generalized linear model (GLM) with the reproductive status of snails (laid eggs/did not lay eggs) as a binomial response variable. We included temperature (15°C, 25°C) and mating type (produced through outcrossing, produced through forced self-fertilization, produced through self-fertilization despite access to a mate) as fixed factors and block (three blocks) as a random factor. In the case of the block, we included only its main effect since the aim was to reduce possible noise in the data that could arise from examining the snails on different days. Since there was no variation among individuals in their reproductive status within some families as well as in one combination of temperature, mating type and population (i.e. all individuals laid eggs), we did not include family and population as factors into the model. We analysed the variation in the number of eggs laid by the snails that oviposited using an analysis of variance (ANOVA) with temperature, mating type and population (two populations) as fixed factors. Additionally, we included family (nested within population-by-mating type interaction; three to seven families per population by mating type combination) and block as random factors. To meet the assumptions of ANOVA, we square root transformed the response variable.

To examine the variation in snail size and immune parameters (PO-like activity and antibacterial activity) we performed separate ANOVAs using similar models as above. To meet the assumptions of ANOVA, we ln-transformed PO-like activity. When we found a statistically significant interaction between mating type and temperature or population, we further analysed the effect of mating type separately for each level of the interacting factor. If a statistically significant effect of mating type was observed, we conducted pairwise comparisons among different categories using specific contrasts. We excluded snails that did not reproduce (11.5%) from the analyses for size and immune defence. This was because such individuals are typically at the end of their lifespan and perform generally poorly (J. Jokela, personal observations). Most importantly, the reproductive potential of our study species is very high, which makes a one week period without laying eggs unlikely in individuals that are not too old. Additionally, at the end of the experiment, snails that did not lay any eggs were 5.2% smaller compared with those that reproduced (independent samples *t*-test: *t*_793_ = -5.121, *p* < 0.001), which indicates slower growth rate. Therefore, these individuals may not represent the snail populations well. We performed all the analyses using IBM SPSS Statistics Version 23.0 (Armonk, NY: IBM Corp.).

## Results

**Fig 1:**
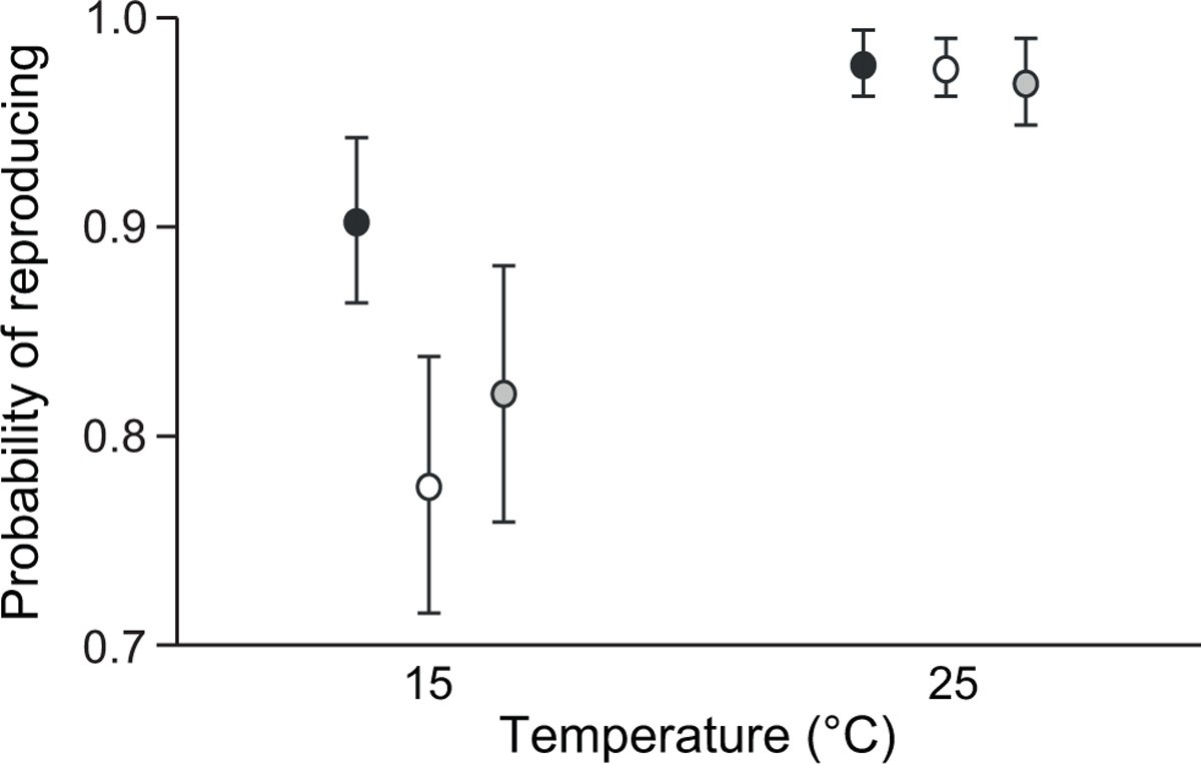
Probability of reproducing in *L*. *stagnalis* snails. Estimated marginal mean (± SE) in individuals produced through outcrossing (black symbols), forced self-fertilization (white symbols) and self-fertilization despite access to a mate (grey symbols)], and maintained at two temperatures (15°C, 25°C) for seven days. Data are not presented separately for different populations since population could not be included in the model as a factor owing to lack of variation in a certain factor combination (see methods section).

**Fig 2:**
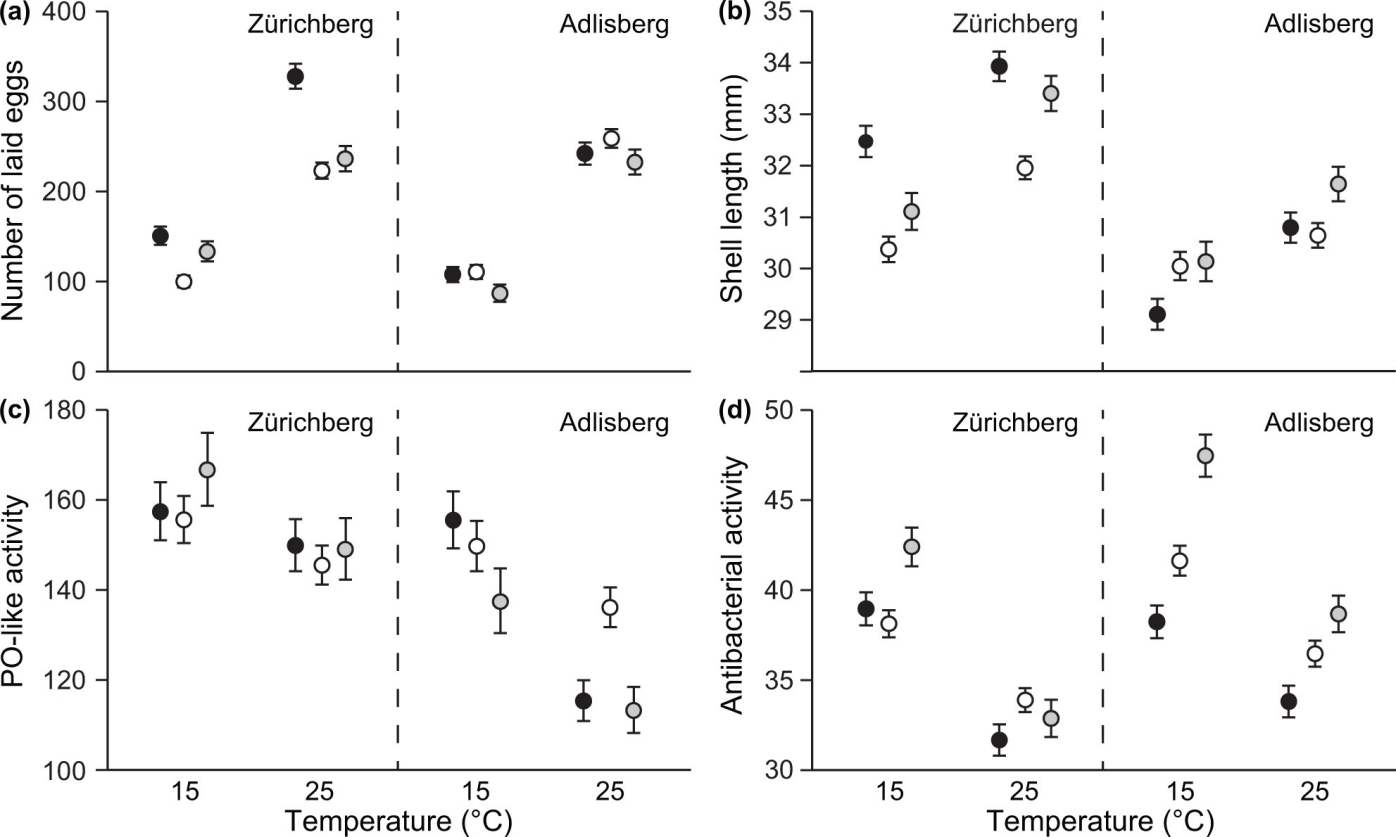
Performance of *L*. *stagnalis* snails in experimental treatments. (A) Number of laid eggs, (B) shell length (mm), (C) phenoloxidase (PO)-like activity [change in OD (range 0–4) in milliunits], and (D) antibacterial activity [change in OD (range 0–4) in milliunits] in individuals produced through outcrossing (black symbols), forced self-fertilization (white symbols) and self-fertilization despite access to a mate (grey symbols)], and maintained at two temperatures (15°C, 25°C) for seven days. Error bars show estimated marginal means ± SE. Data are shown separately for the Zürichberg and the Adlisberg populations.

Snails showed typical responses to the experimental heat wave. First, exposure to high temperature increased their probability to reproduce (17.0% increase compared with 15°C; GLM: d.f. = 1, *F* = 30.045, *p* < 0.001; Fig 1), the number of laid eggs (123.1% increase; Table 1 and Fig 2), and size (4.7% increase; Table 1 and Fig 2). Second, immune activity was reduced by the high temperature (11.6% reduction in PO-like activity, 14.8% reduction in antibacterial activity; Table 1 and Fig 2).

**Table 1:** Analyses of variance for the number of laid eggs, shell length, PO-like activity, and antibacterial activity of *L*. *stagnalis* snails.

Trait	Factor	df	MS	*F*	η^2^(%)	*p*
Number of eggs	**Temperature (T)**	**1**	**4351.442**	**494.348**^**1**^	**36.2**	**< 0.001**
	Mating type (M)	2	108.864	2.414^2^	1.8	0.114
	Population (P)	1	105.071	2.333^2^	0.9	0.142
	**Family (F) (M × P)**	**21**	**45.028**	**5.115**^**1**^	**7.9**	**< 0.001**
	Block	2	20.871	2.377	0.3	0.094
	T × M	2	4.447	0.505^**1**^	0.1	0.611
	T × P	1	18.265	2.075^**1**^	0.2	0.164
	**M × P**	**2**	**161.556**	**3.582**^2^	**2.7**	**0.046**
	T × F (M × P)	21	8.802	1.003	1.5	0.458
	T × P × M	2	21.502	2.443^**1**^	0.4	0.111
	Error	657	8.780			
Shell length	**Temperature (T)**	**1**	**366.880**	**103.868**^**1**^	**7.1**	**< 0.001**
	Mating type (M)	2	58.354	2.278^2^	2.3	0.127
	**Population (P)**	**1**	**522.591**	**20.428**^2^	**10.1**	**< 0.001**
	**Family (F) (M × P)**	**21**	**25.576**	**7.239**^**1**^	**10.4**	**< 0.001**
	**Block**	**2**	**57.273**	**11.844**	**2.2**	**< 0.001**
	T × M	2	9.270	2.626^**1**^	0.4	0.096
	T × P	1	11.085	3.138^**1**^	0.2	0.091
	**M × P**	**2**	**99.066**	**3.867**^2^	**3.8**	**0.037**
	T × F (M × P)	21	3.533	0.731	1.4	0.803
	T × P × M	2	6.357	1.801^**1**^	0.2	0.190
	Error	657	4.836			
PO-like activity	**Temperature (T)**	**1**	**2.920**	**13.117**^**1**^	**3.6**	**0.002**
	Mating type (M)	2	0.103	0.198^2^	0.3	0.822
	**Population (P)**	**1**	**3.045**	**5.861**^2^	**3.7**	**0.025**
	**Family (F) (M × P)**	**21**	**0.519**	**2.334**^**1**^	**13.3**	**0.029**
	Block	2	0.149	1.694	0.4	0.185
	T × M	2	0.155	0.695^**1**^	0.4	0.510
	T × P	1	0.567	2.549^**1**^	0.7	0.125
	M × P	2	0.431	0.829^2^	1.1	0.450
	**T × F (M × P)**	**21**	**0.223**	**2.528**	**5.7**	**< 0.001**
	T × P × M	2	0.205	0.918^**1**^	0.5	0.415
	Error	657	0.088			
Antibacterial activity	**Temperature (T)**	**1**	**6819.664**	**201.638**^**1**^	**14.1**	**< 0.001**
	Mating type (M)	2	987.342	2.872^2^	4.1	0.079
	Population (P)	1	1470.768	4.285^2^	3.0	0.051
	**Family (F) (M × P)**	**21**	**343.119**	**10.143**^**1**^	**14.9**	**< 0.001**
	Block	2	94.993	2.172	0.4	0.115
	**T × M**	**2**	**258.446**	**7.645**^**1**^	**1.1**	**0.003**
	T × P	1	29.928	0.885^**1**^	0.1	0.358
	M × P	2	253.937	0.739^2^	1.1	0.490
	T × F (M × P)	21	33.826	0.773	1.5	0.754
	T × P × M	2	58.413	1.728^**1**^	0.2	0.202
	Error	657	43.741			

^1^ T × F(M × P) as the error term

^2^ F(M × P) as the error term

Factors are water temperature (15°C, 25°C), mating type (produced through outcrossing, produced through forced self-fertilization, produced through self-fertilization despite access to a mate), population (2 populations), family (3–7 families per mating type per population), and block (3 blocks). The effect size η^2^ shows the proportion of total variance explained by each factor. Statistically significant effects are in bold.

The above effects of temperature were largely independent of the snail mating type. Inbreeding affected only the effect of temperature on antibacterial activity indicated by a significant temperature-by-mating type interaction (Table 1). However, this interaction explained only 1.1% of the total variance in that trait (Table 1). Furthermore, the observed effect was because at 15°C, snails produced through self-fertilization despite access to a mate had 13.0–16.4% higher antibacterial activity compared with the other two groups (ANOVA: *F*_2,21_ = 5.997, *p* = 0.009; pairwise contrasts: contrast estimate ≤ -4.949, *p* < 0.001 for both; Fig 2), but there was no effect of mating type on antibacterial activity at 25°C (ANOVA: *F*_2,21_ = 1.222, *p* = 0.315; Fig 2). Thus, the interactive effect was not because of the poor performance of those snails at high temperature. In other examined traits, temperature-by-mating type interaction explained ≤ 0.4% of the total variance (Table 1).

Additionally, the two examined populations differed in the effect of mating type on the number of laid eggs and snail size indicated by significant mating type-by-population interactions (Table 1). This was because, in the Zürichberg population, snails produced through outcrossing were 3.0–6.5% larger and laid 27.5–58.5% more eggs compared with the other two groups (ANOVA: *F*_2,11_ ≥ 5.143, *p* ≤ 0.027 for both; pairwise contrasts: contrast estimate ≤ -0.944, *p* ≤ 0.004 for all; Fig 2). On the other hand, snails produced through forced self-fertilization were smallest and laid fewest eggs (pairwise contrasts: contrast estimate ≤ -0.988, *p* ≤ 0.014 for all; Fig 2). In the Adlisberg population, mating type did not affect snail size or reproductive output (ANOVA: *F*_2,10_ ≤ 0.826, *p* ≥ 0.466; Fig 2). All tested traits showed family-level variation (Table 1). In PO-like activity, families also differed in their responses to the temperature indicated by a significant temperature-by-family interaction (Table 1).

## Discussion

The increasing frequency and severity of summer heat waves [1, 51] affect natural populations of many species [52–54]. Organisms’ responses to environmental change can, however, depend on their genetic properties such as the level of inbreeding [22–24]. In our study, exposure of *L*. *stagnalis* snails to an experimental heat wave (25°C versus 15°C) increased snail reproduction and size but reduced their immune function. Life history traits were negatively affected by inbreeding in one of the studied populations, but this effect was independent of temperature. Snails produced through self-fertilization despite access to a mate showed the strongest decrease in antibacterial activity under high temperature. This was, however, unlikely to be due to inbreeding because they did not show reduced defence at 25°C, but the highest enzyme activity at 15°C.

The above effects of high temperature on snails are in line with our previous findings and may be due to altered resource allocation among traits or due to lack of, for instance, micronutrients required for repair mechanisms [30]. Reduction in reproduction and size in inbred snails in one of the study populations indicates that inbreeding can have negative effects on life history traits in *L*. *stagnalis* (see also [28]), but this effect may vary across populations. In earlier studies, *L*. *stagnalis* and other hermaphroditic species have not shown severe consequences of inbreeding [29, 37, 55]. In general, frequent inbreeding is expected to purge the genetic load owing to recessive deleterious alleles (i.e. select against them) and to reduce fitness losses associated with inbreeding [16]. This is the case especially when natural selection is strong [16]. However, *L*. *stagnalis* prefers outcrossing in the wild [37, 38], which could reduce the effect of purging.

Inbreeding did not alter the responses of snails to high temperature in most of the examined traits. Thus, heat waves may not induce/intensify the negative effects of inbreeding in our study species. This could be due to two different reasons. First, previously experienced environmental changes may have induced selection against recessive deleterious alleles that reduce snail fitness [13, 56]. Second, our experimental treatments may not have been strong enough to induce such effects. Snails produced through self-fertilization despite access to a mate showed the strongest reduction in antibacterial activity under high temperature. This was because of the highest enzyme activity in them compared with other mating types at 15°C. Hence, the effect was unlikely to be due to inbreeding depression. The reason for this effect is unknown but there are different possible explanations. First, individuals that self-fertilized in the presence of a mate may have been highly self-compatible leading to exceptionally fit offspring [57, 58], or they may have had high physiological condition making them choosy for potential mates. Second, their mates may have been immunologically challenged, for example, by opportunistic microorganisms leading to a refusal to mate and cross-generational immune priming to increase the immune activity of the offspring [59, 60].

We found high family-level variation in all examined traits at both temperatures. We also detected a family-by-temperature (i.e. G × E) interaction affecting the PO-like activity of snail haemolymph. These results are in line with our earlier findings showing abundant genetic variation in immune and life history traits under similar temperature conditions and in the response of PO-like activity to high temperature [15]. This indicates genetic potential for adaptation in both thermal environments, and that the cost of exposure to high temperature is lower for some families and can be selected for.

To conclude, *L*. *stagnalis* snails exposed to high temperature increased reproduction and size, but reduced immune activity. Furthermore, inbreeding (i.e. self-fertilization) reduced reproductive output in one of the two examined populations. However, the effects of high temperature were not affected by inbreeding. This may be due to previous purging, which is most efficient in frequently inbreeding populations exposed to intense selective pressure [16]. Our results indicate that inbreeding can have negative effects on the performance of *L*. *stagnalis* but environmental changes that lead to more frequent self-fertilization and/or biparental inbreeding, for example, because of reduced population size may not change the responses of snails to heat waves.

## Supporting information

S1 Table Results of the genetic analyses of parental snails. Polymorphism data, inbreeding coefficients and selfing rate estimates for both laboratory stock populations (Zürichberg, Adlisberg) based on the analyses on parental snails used to produce outcrossed families. Loci B117, 2k46, B4, and A112 were removed because of the difficulties in scoring the peaks.
(XLSX)
